# Longitudinal single-cell analysis identifies a chemotherapy-suppressed proliferative program associated with adverse prognosis in neuroblastoma

**DOI:** 10.3389/fgene.2026.1878093

**Published:** 2026-10-02

**Authors:** Aiguo Zhu, Jian Wang

**Affiliations:** 1 Tianjin Cancer Hospital Airport Hospital, National Clinical Research Center for Cancer, Tianjin, China; 2 Key Laboratory of Cancer Prevention and Therapy, Tianjin’s Clinical Research Center for Cancer, National Clinical Research Center for Cancer, Tianjin Medical University Cancer Institute and Hospital, Tianjin, China

**Keywords:** chemotherapy, MYC, neuroblastoma, prognosis, proliferative program, single-cell RNA sequencing

## Abstract

**Background:**

High-risk neuroblastoma frequently relapses after intensive therapy, but the treatment-associated malignant-neuroblast programs that inform baseline tumor risk remain incompletely defined. We used longitudinal single-cell/single-nucleus RNA-seq data to identify post-treatment transcriptional changes in malignant neuroblasts and evaluated their prognostic relevance in diagnostic bulk RNA-seq cohorts.

**Methods:**

We analyzed a longitudinal single-cell/single-nucleus RNA-seq atlas of diagnostic (DX) and post-treatment (PTX) neuroblastoma samples. Neuroblast cells were aggregated into donor- and treatment-state-level pseudo-bulk profiles, and paired differential expression analysis was performed to identify PTX-regulated malignant-neuroblast programs. PTX-down and PTX-up gene sets were functionally characterized by Hallmark over-representation analysis and Hallmark pathway activities were further estimated by GSVA in bulk cohorts. We then projected the PTX-down program onto diagnostic bulk RNA-seq cohorts by calculating a PTX-down proliferation score as the mean cohort-wise z-score of PTX-down genes. Prognostic value was evaluated in a primary diagnostic bulk RNA-seq validation cohort and three independent external diagnostic bulk RNA-seq cohorts. Clinical model increment, cross-cohort meta-analysis, clinical feature association, and Hallmark correlation analyses were performed.

**Results:**

Paired pseudo-bulk analysis showed that PTX malignant-neuroblast profiles had reduced activity of a proliferative-replication program enriched for E2F targets, G2/M checkpoint, MYC targets, DNA repair, and mitotic spindle pathways, together with relative enrichment of adaptive-state features related to hypoxia, inflammatory signaling, apoptosis, and epithelial-mesenchymal transition. In the primary validation cohort, high PTX-down proliferation score was associated with inferior OS and EFS and remained prognostic after adjustment for age, COG-defined high-risk classification, MYCN status, and INSS stage. Adding the PTX-down score improved C-index and 1-, 3-, and 5-year time-dependent AUC beyond both COG and clinical models. Across four bulk RNA-seq cohorts, higher PTX-down score was consistently associated with adverse OS (random-effects pooled HR = 1.83, 95% CI 1.20–2.80; P = 0.005). The score was also higher in tumors with MYCN amplification, stage 4 disease, and age ≥ 18 months. Core-gene and Hallmark correlation analyses defined the PTX-down score as an E2F/G2M/MYC-driven proliferative axis.

**Conclusion:**

Longitudinal single-cell analysis identifies a chemotherapy-suppressed proliferative program in malignant neuroblasts. Projection of this program onto diagnostic bulk tumors captures high-risk biology, improves prognostic discrimination, and predicts adverse outcome in neuroblastoma.

## Introduction

Neuroblastoma is the most common extracranial solid tumor of childhood and remains a major cause of cancer-related mortality in pediatric oncology (Maris et al., 2007). Although multimodal treatment has improved outcomes for selected patients, high-risk neuroblastoma continues to be characterized by relapse and therapeutic resistance (Matthay et al., 1999; Yu et al., 2010). Tumor recurrence after intensive therapy suggests that malignant neuroblasts undergo profound therapy-associated remodeling and that treatment-shaped transcriptional programs may contain clinically relevant information about tumor aggressiveness (Cohn et al., 2009; Pinto et al., 2015).

The biological heterogeneity of neuroblastoma has been extensively linked to neural crest development, developmental cell states, MYCN activation, proliferative signaling, chromatin regulation, immune microenvironmental features, and lineage plasticity (Johnsen et al., 2019). Genomic and transcriptomic studies have established that aggressive neuroblastoma is shaped by recurrent high-risk alterations and transcriptional state diversity, including MYCN amplification, structural chromosomal alterations, telomere-maintenance mechanisms, and developmental plasticity (Cheung and Dyer, 2013). Recent single-cell, single-nucleus, multi-omic, and spatial transcriptomic studies have further shown that neuroblastoma contains diverse malignant and non-malignant compartments, with malignant neuroblasts occupying states related to adrenergic (ADRN) identity, mesenchymal (MES) or stress-associated programs, proliferative activity, and developmental plasticity (Jansky et al., 2021; Xu et al., 2025). However, most prognostic transcriptomic analyses remain based on diagnostic bulk tumor profiles. These bulk analyses can identify outcome-associated programs, but they often lack direct information about how tumor-cell states change after treatment. Consequently, the relationship between chemotherapy-associated malignant-neuroblast remodeling and baseline prognostic biology remains incompletely understood.

Longitudinal single-cell or single-nucleus RNA-seq datasets provide a unique opportunity to directly compare malignant neuroblasts before and after treatment. Recent longitudinal profiling of high-risk neuroblastoma before and after induction chemotherapy has demonstrated substantial therapy-associated rewiring of both tumor and microenvironmental compartments, with poor outcome linked to proliferative and metabolically active neoplastic states and more differentiated neuronal-like states linked to more favorable biology (Yu et al., 2025). By focusing on paired diagnostic and post-treatment tumor-cell profiles, it is therefore possible to identify transcriptional programs that are suppressed or retained after induction therapy. Such programs may reflect therapy-sensitive proliferative states, residual adaptive states, or stress-associated phenotypes. Importantly, if a program suppressed after treatment is highly active at diagnosis, it may mark tumors with high baseline proliferative capacity and aggressive clinical behavior. Thus, therapy-associated single-cell programs may serve not only as descriptors of post-treatment remodeling but also as biologically interpretable markers of diagnostic tumor risk (Xu et al., 2025).

In this study, we analyzed a longitudinal single-cell/single-nucleus RNA-seq atlas of neuroblastoma to define chemotherapy-associated transcriptional programs in malignant neuroblasts. Neuroblast cells were aggregated into paired pseudo-bulk profiles by donor and treatment state, enabling donor-aware comparison between diagnostic (DX) and post-treatment (PTX) samples. This analysis identified a PTX-down program enriched for E2F targets, G2/M checkpoint, MYC targets, DNA repair, and mitotic spindle pathways, together representing a chemotherapy-suppressed proliferative-replication axis. In parallel, PTX-up genes were enriched for hypoxia, inflammatory signaling, apoptosis, and epithelial-mesenchymal transition-related programs, consistent with therapy-associated adaptive states.

We then projected the PTX-down program onto diagnostic bulk RNA-seq cohorts as a PTX-down proliferation score and evaluated its clinical relevance across multiple neuroblastoma datasets. In GSE62564, the score predicted adverse overall survival and event-free survival, remained informative after adjustment for clinical risk variables, and improved prognostic discrimination beyond COG-defined high-risk classification and conventional clinical models. External validation across three independent diagnostic bulk RNA-seq cohorts further supported its association with adverse overall survival and high-risk clinical features. Finally, core-gene and Hallmark correlation analyses demonstrated that the PTX-down score reflects an E2F/G2M/MYC-driven proliferative axis in diagnostic tumors. Together, these findings establish a longitudinal single-cell-derived, biologically interpretable program that links chemotherapy-suppressed malignant-neuroblast proliferation to baseline high-risk biology and adverse prognosis.

## Methods

### Data sources and cohort inclusion

The discovery dataset was obtained from the published longitudinal single-cell/single-nucleus RNA-seq neuroblastoma atlas by Yu et al., which profiled diagnostic (DX) and post-treatment (PTX) neuroblastoma samples (Yu et al., 2025). This atlas was used in the present study to identify therapy-associated transcriptional changes within the malignant-neuroblast compartment. The preprocessed single-cell/single-nucleus RNA-seq object and accompanying cell-level annotations were used as the basis for malignant-neuroblast selection, donor-treatment pseudo-bulk aggregation, and paired PTX versus DX differential expression analysis. Bulk RNA-seq cohorts were then used for diagnostic-cohort validation. These validation cohorts were selected *a priori* based on four criteria: publicly available neuroblastoma diagnostic transcriptomic data, harmonizable gene-level expression profiles, available survival information, and sufficient clinical annotation for risk-related analyses.

GSE62564 (n = 498) was designated as the primary validation cohort because it had the largest matched expression-clinical sample size in this study and contained complete OS and EFS information, allowing Kaplan-Meier analysis, Cox regression, clinical adjustment, C-index comparison, time-dependent AUC analysis, and Hallmark bridge analysis. GSE181559 (n = 97), TARGET-NBL (n = 161), and the fwr144 cohort (n = 144) (Zhang et al., 2015; Wei et al., 2018; Hagemann et al., 2023) were included as independent external validation cohorts to assess the cross-cohort robustness of the PTX-down proliferation score across datasets generated from different sources and preprocessing backgrounds. The role of each dataset, expression processing strategy, available clinical variables, and corresponding analyses are summarized in Supplementary Table S1.

### Single-cell atlas processing and malignant-neuroblast pseudo-bulk generation

The longitudinal single-cell/single-nucleus RNA-seq dataset was obtained as a preprocessed h5ad object containing cell-level annotation, treatment state, donor information, and low-dimensional embeddings. We used the curated cell-type and neoplastic-cell annotations provided by the original atlas. In that study, neoplastic cell calls were supported by inferCNV-based detection of recurrent neuroblastoma-associated copy-number alterations, including gains of 17q and losses of 1p/11q. We did not perform *de novo* malignant-cell calling. Instead, cells annotated as neoplastic/malignant neuroblasts in the source atlas were selected as the malignant-neuroblast compartment for downstream analysis. Non-malignant compartments, including immune cells, endothelial cells, fibroblasts, Schwann cells, adrenal cortical cells, hepatocytes, and kidney-related cells, were excluded before pseudo-bulk aggregation.

Cell-type labels from the source atlas were harmonized only at the nomenclature level for visualization and summary plotting; no *de novo* batch correction, cell integration, or re-clustering was performed in this study. The precomputed low-dimensional embeddings provided in the h5ad object were used for visualization of global cellular composition and treatment-state distribution. Single-cell data handling, annotation subsetting, and visualization were performed in Python using Scanpy/AnnData together with pandas, NumPy, and Matplotlib (Wolf et al., 2018). UMAP plots were generated from the atlas-provided embeddings to inspect cell-type composition, treatment-state distribution, and the selected malignant-neuroblast compartment. To reduce cell-level sparsity and enable donor-aware comparison, the selected malignant neuroblast cells were aggregated into pseudo-bulk profiles according to donor and treatment state. Raw counts from cells belonging to the same donor-treatment combination were summed to generate malignant-neuroblast pseudo-bulk count profiles. Donor-treatment groups containing at least 20 malignant neuroblast cells were retained, and paired differential expression analysis was restricted to 22 donors with matched DX and PTX pseudo-bulk profiles, yielding 44 malignant-neuroblast pseudo-bulk profiles for downstream modeling of PTX versus DX transcriptional changes.

MDS visualization of malignant-neuroblast pseudo-bulk profiles was performed using the plotMDS function in edgeR/limma. The pseudo-bulk count matrix was converted to log-counts-per-million internally for MDS visualization. The axes labeled “leading logFC dimensions” represent the leading log2-fold-change distances used by the MDS algorithm to summarize pairwise sample-to-sample expression differences. These leading logFC dimensions are distinct from the PTX versus DX differential expression logFC estimates and were used only for exploratory visualization, not for defining PTX-down or PTX-up genes.

### Paired pseudo-bulk differential expression analysis

Differential expression analysis between PTX and DX malignant-neuroblast pseudo-bulk profiles was performed using edgeR. Raw pseudo-bulk count matrices were used to construct a DGEList object. Lowly expressed genes were filtered using the design-aware edgeR filterByExpr function with min.count = 10, and normalization factors were calculated using the trimmed mean of M-values method. Biological coefficient of variation and multidimensional scaling plots were inspected to evaluate dispersion structure and sample-level relationships (Robinson et al., 2010).

A paired design was used to account for donor-specific effects. Differential expression was modeled with treatment state as the main variable of interest and donor pairing included in the design matrix. Genes with FDR < 0.05 and positive log2 fold change in PTX versus DX were defined as PTX-up genes, whereas genes with FDR < 0.05 and negative log2 fold change were defined as PTX-down genes. In total, 1,601 PTX-up and 1,478 PTX-down genes were identified at FDR < 0.05 after expression filtering. Full differential expression statistics, including log2 fold change, test statistics, nominal P values, and adjusted P values, are provided in Supplementary Table S2.

### Definition of PTX-up and PTX-down gene sets

To prioritize representative therapy-regulated genes, significant PTX-up and PTX-down genes were ranked using a composite score defined as |log2 fold change| × -log10(FDR). This metric was selected to balance the magnitude of treatment-associated expression change and statistical confidence from the paired pseudo-bulk differential expression analysis. The top 300 PTX-down and top 300 PTX-up genes were used in the primary analysis because this gene-set size provided a practical balance between stable score estimation and specificity of the treatment-associated signal. These gene sets are provided in Supplementary Table S3.

To assess the robustness of this choice, sensitivity analyses were performed using alternative gene-set sizes, including the top 200 and top 400 genes. In addition, an alternative ranking strategy based on absolute log2 fold change alone was evaluated. For each alternative gene set, PTX-derived scores were recalculated in bulk RNA-seq cohorts using the same within-cohort gene-wise z-score strategy, and their prognostic associations, correlation with the primary PTX-down score, and Hallmark enrichment patterns were assessed.

### Hallmark enrichment analysis of PTX-regulated gene sets

Hallmark pathway over-representation analysis was performed for PTX-down and PTX-up gene sets using MSigDB Hallmark gene sets (Liberzon et al., 2015). The background universe was defined as the set of genes retained after expression filtering in the paired pseudo-bulk analysis. Enrichment was evaluated using a hypergeometric framework, and multiple testing correction was performed using the Benjamini–Hochberg method. Enrichment ratio, gene count, and adjusted P value were used for visualization. For visualization purposes only, −log10(FDR) values exceeding 50 were capped at 50 in dot plots; original values are provided in Supplementary Table S7.

### Bulk RNA-seq preprocessing and score calculation

Bulk RNA-seq expression matrices were processed according to cohort-specific formats. Gene symbols were harmonized across datasets, and duplicated gene symbols were collapsed where necessary. Expression values were log-transformed or used as preprocessed according to the data format of each cohort, as summarized in Supplementary Table S1. For score calculation, only PTX-down or PTX-up genes available in each bulk RNA-seq cohort were retained.

Within each bulk cohort, gene expression values were standardized gene-wise across samples. For gene g in sample i, the standardized expression value was calculated as:

$$z_{gi}=\frac{x_{gi}-\mu_{g}}{\sigma_{g}}$$

where *x*_*gi*_ is the expression value of gene g in sample i, and *μ*_*g*_ and *σ*_*g*_ represent the mean and standard deviation of gene g across samples within the same cohort.

The PTX-down proliferation score for sample i was calculated as the mean standardized expression of retained PTX-down genes:

$${PTX‐down\;score}_{i}=\frac{1}{n_{down}}\sum_{g\in G_{down}}z_{gi}$$

where G_down_ represents the set of PTX-down genes retained in the corresponding cohort, and n_down_ is the number of retained PTX-down genes.

The PTX-up adaptation score was calculated analogously:

$${PTX‐up\;score}_{i}=\frac{1}{n_{up}}\sum_{g\in G_{up}}z_{gi}$$

where G_up_ represents the set of retained PTX-up genes, and n_up_ is the number of retained PTX-up genes.

The integrated score was calculated as:

$${Integrated\;score}_{i}={PTX‐up\;score}_{i}-{PTX‐down\;score}_{i}$$

Random gene-set null analysis was performed in GSE62564 to evaluate whether the prognostic association of the PTX-down proliferation score was specific to the PTX-down gene set. We randomly sampled 1,000 gene sets of the same size as the primary PTX-down top300 gene set from the expressed gene background. For each random gene set, a random gene-set score was calculated using the same within-cohort gene-wise z-score strategy as the PTX-down proliferation score. Cox regression was then performed for OS and EFS per 1-SD increase in each random score. The empirical one-sided P value was calculated as the proportion of random gene sets with a Cox logHR equal to or greater than that of the primary PTX-down top300 score.

### Survival analysis in GSE62564

GSE62564 was used as the primary bulk validation cohort. Overall survival (OS) and event-free survival (EFS) were analyzed according to available clinical annotation. Kaplan-Meier curves were generated to compare survival between PTX-down high and low groups, and log-rank tests were used to assess survival differences. Cox proportional hazards models were used to estimate hazard ratios and 95% confidence intervals.

For continuous-score analyses, PTX-derived scores were scaled to 1 standard deviation before Cox regression. Multivariable Cox models were adjusted for age, COG-defined high-risk classification, MYCN status, and INSS stage. Cox regression results and related sensitivity analyses are provided in Supplementary Table S4.

### Clinical model comparison and time-dependent AUC analysis

To evaluate whether the PTX-down proliferation score improved prognostic discrimination beyond clinical variables, two baseline models were constructed in GSE62564. The first model was based on COG-defined high-risk classification. The second clinical model included age, MYCN status, and INSS stage. For each baseline model, a corresponding expanded model was generated by adding the PTX-down proliferation score.

Model performance was assessed using the C-index and time-dependent AUC. Time-dependent AUC was evaluated at 1, 3, and 5 years for OS and EFS using the IPCW-based timeROC framework (Blanche et al., 2013). To estimate uncertainty for model-performance comparisons, 95% confidence intervals for the C-index and time-dependent AUC values were calculated using patient-level bootstrap resampling. Cox models were fitted in the original GSE62564 dataset to generate model-specific linear predictors. In each bootstrap iteration, patients were resampled with replacement, and C-index and IPCW-based time-dependent AUC values at 1, 3, and 5 years were recalculated using the corresponding model linear predictors. Confidence intervals were defined using the 2.5th and 97.5th percentiles of the bootstrap distribution. Bootstrap confidence intervals for model-performance differences were calculated from paired differences between the expanded model and the corresponding baseline model within each bootstrap resample. AUC improvement was calculated as the AUC of the expanded model minus the AUC of the corresponding baseline model. Original model increment metrics are provided in Supplementary Table S5, and bootstrap confidence intervals and model-performance differences are provided in Supplementary Table S9.

### Cross-cohort survival validation and meta-analysis

The association between PTX-down proliferation score and OS was evaluated across GSE62564, GSE181559, TARGET-NBL, and fwr144. Within each cohort, Cox regression was performed using the PTX-down score as a continuous variable scaled to 1 standard deviation. Hazard ratios and standard errors were extracted for cross-cohort synthesis.

Random-effects meta-analysis was performed using restricted maximum likelihood estimation. The pooled hazard ratio and 95% confidence interval were estimated across cohorts, and between-cohort heterogeneity was assessed using Cochran’s Q, tau-squared, and the I-squared statistic. Cohort-specific Cox results and meta-analysis outputs are provided in Supplementary Table S6.

### Clinical feature association analysis

To evaluate whether the PTX-down proliferation score was associated with established high-risk clinical features, median score differences were calculated between high-risk and reference subgroups within each cohort. The evaluated clinical features included MYCN amplification, stage 4 disease, and age 18 months or older. For each feature, the score difference was defined as the median PTX-down score in the high-risk subgroup minus the median score in the corresponding reference subgroup. Positive values indicated higher PTX-down score in clinically high-risk tumors.

### Hallmark pathway scoring and correlation analysis

To interpret the biological meaning of PTX-derived scores in diagnostic bulk tumors, Hallmark pathway activities were estimated in GSE62564 using GSVA. Selected Hallmark pathways related to proliferation, DNA replication, mitotic regulation, stress response, hypoxia, apoptosis, inflammatory signaling, and epithelial-mesenchymal transition were analyzed. GSVA was applied to log-transformed continuous expression values, with the gene set size filter set to 10–500 genes (all selected Hallmark gene sets met this criterion) (Hänzelmann et al., 2013). Spearman correlation coefficients were calculated between PTX-derived scores and Hallmark pathway activity scores. Correlation matrices were visualized as heatmaps. Hallmark enrichment and correlation results are provided in Supplementary Table S7.

### Core PTX-down gene bridge analysis

Representative core PTX-down genes were selected to reflect major functional components of the chemotherapy-suppressed proliferative program, including the E2F/MYC axis, DNA replication, and cell-cycle/mitotic regulation. In GSE62564, samples were ordered by PTX-down proliferation score, and expression values of core genes were visualized as row-scaled z-scores. Clinical annotations, including PTX-down score group, COG-defined high-risk classification, MYCN status, and INSS stage, were added to connect core gene expression patterns with high-risk clinical features.

### Statistical analysis

All statistical analyses were performed using R (version 4.2.2) and Python (version 3.11.3). Differential expression analysis was performed using edgeR (version 3.40.2). Survival analyses were conducted using the survival (v3.8.6), survminer (v0.4.9), and timeROC (v0.4) packages. Enrichment analysis P values were adjusted using the Benjamini–Hochberg method. Unless otherwise specified, two-sided P values were reported, and P < 0.05 was considered statistically significant. Visualization was performed using R packages including ggplot2, pheatmap, survminer, timeROC, and tidyverse packages, and Python packages including scanpy, pandas, numpy, and matplotlib.

## Results

### Longitudinal single-cell profiling defines the malignant-neuroblast compartment for paired pseudo-bulk analysis

To investigate therapy-associated transcriptional remodeling in neuroblastoma, we analyzed a longitudinal single-cell/single-nucleus RNA-seq atlas containing diagnostic (DX) and post-treatment (PTX) samples. UMAP visualization of the full atlas showed multiple annotated cell populations, including neuroblast cells, immune cells, stromal cells, endothelial cells, Schwann cells, adrenal cortical cells, hepatocytes, and kidney-related cells, indicating that the dataset captured both tumor and non-tumor compartments within the sampled specimens (Figure 1A). Because the central aim of this study was to identify therapy-associated tumor-cell programs, subsequent analyses were restricted to cells annotated as neoplastic/malignant neuroblasts in the source atlas. Non-malignant immune, stromal, endothelial, Schwann, adrenal cortical, hepatic, and kidney-related compartments were excluded before pseudo-bulk aggregation.

**FIGURE 1:**
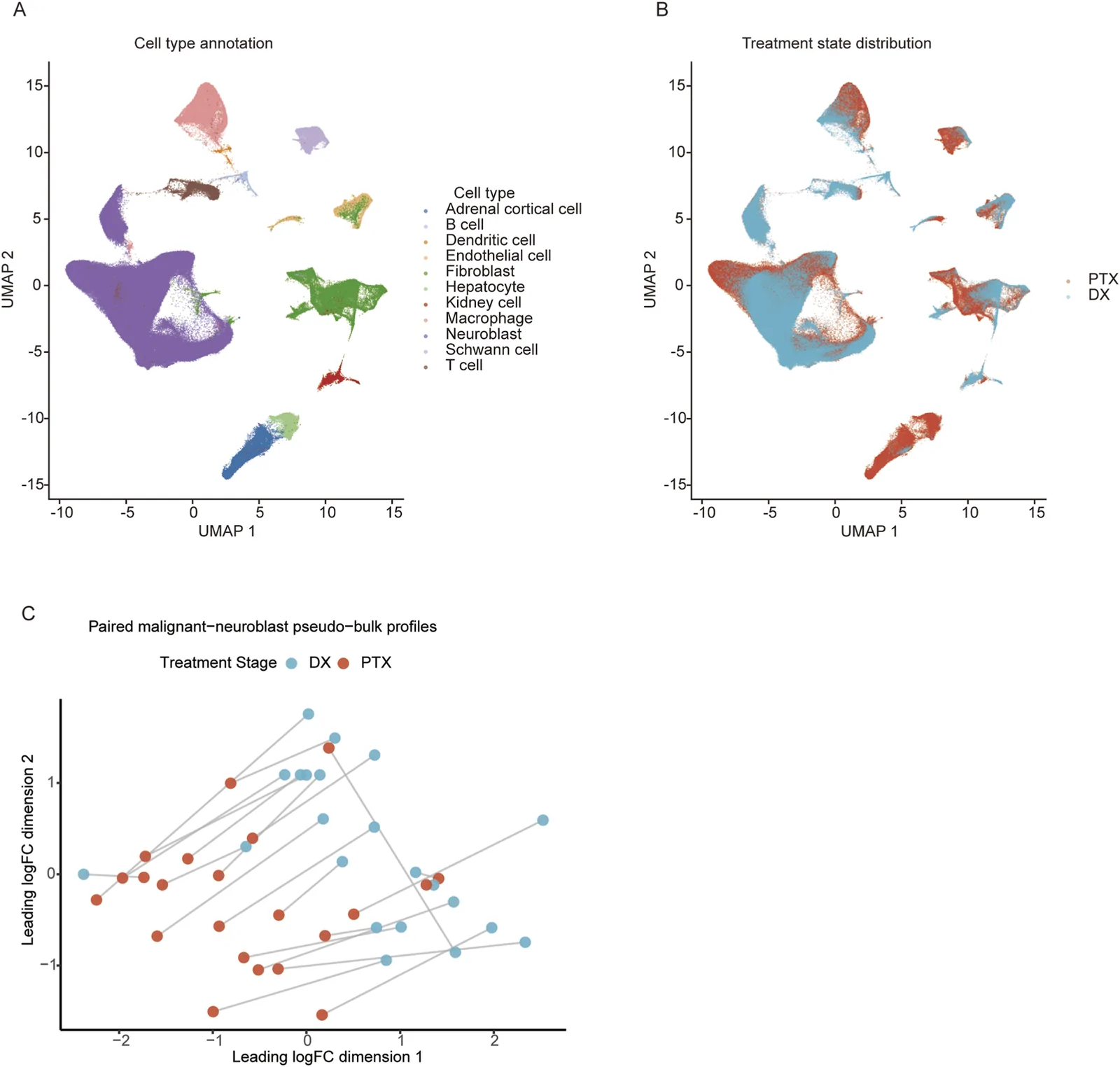
Overview of the longitudinal single-cell atlas and paired pseudo-bulk analysis framework. **(A)** UMAP visualization of the longitudinal single-cell atlas colored by annotated cell type, showing neuroblast cells together with immune, stromal, endothelial, Schwann, adrenal cortical, hepatic, and kidney-related cell populations. **(B)** UMAP visualization of treatment-state distribution across the single-cell atlas, with diagnostic (DX) and post-treatment (PTX) cells highlighted. **(C)** MDS plot of malignant-neuroblast pseudo-bulk profiles generated by donor and treatment state using edgeR/limma. Samples are colored by treatment state, and matched DX/PTX profiles from the same donor are connected to indicate the paired study design. The axes labeled as leading logFC dimensions represent MDS distances based on pairwise sample-to-sample expression differences and are distinct from the PTX versus DX differential expression logFC estimates.

Treatment-state visualization of the full atlas showed that DX and PTX cells were broadly represented across multiple cellular regions, confirming that both time points were captured in the dataset (Figure 1B). We then focused on neuroblast cells and aggregated them into malignant-neuroblast pseudo-bulk profiles by donor and treatment state. This generated 44 pseudo-bulk profiles from 22 matched DX/PTX donors. MDS analysis visualized the global transcriptional distribution of these paired pseudo-bulk profiles and showed treatment-associated separation between DX and PTX profiles (Figure 1C). Paired DX/PTX profiles from the same donor were connected in the plot to indicate the matched study design, rather than to infer patient-specific relationships from the MDS plot alone. This matched donor structure supported the use of a donor-aware paired pseudo-bulk differential expression framework to identify PTX-associated malignant-neuroblast transcriptional changes.

### PTX malignant-neuroblast profiles show reduced proliferative-replication programs and relative enrichment of adaptive-state features

We next performed paired differential expression analysis comparing PTX versus DX malignant-neuroblast pseudo-bulk profiles. This analysis identified 1,478 PTX-down and 1,601 PTX-up genes at FDR < 0.05 (Figure 2A). Among the PTX-down genes, multiple canonical proliferation, cell-cycle, and DNA replication genes were observed, including MKI67, TOP2A, PCNA, MCM2, MCM4, MCM6, MCM10, CDC45, E2F1, UHRF1, BIRC5, and AURKB. In contrast, PTX-up genes included neuroendocrine, stress-response, and adaptive-state-associated genes such as CALCA, ADCYAP1, VIP, DDIT3, HBEGF, NR4A1, and NR4A3 (Figure 2A).

**FIGURE 2:**
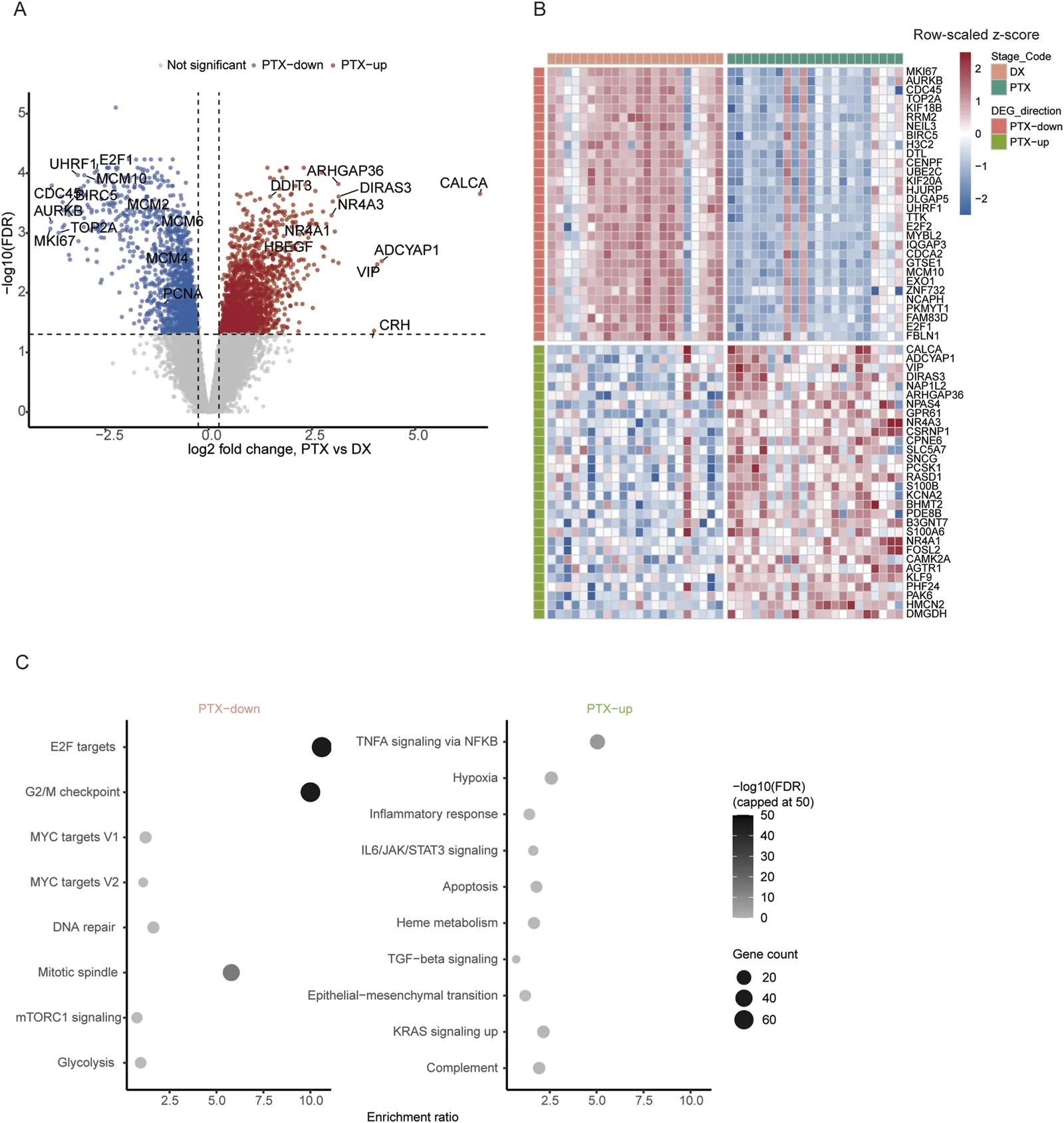
PTX malignant-neuroblast profiles show reduced proliferative-replication programs and relative enrichment of adaptive-state features. **(A)** Volcano plot showing differentially expressed genes between PTX and DX malignant-neuroblast pseudo-bulk profiles. Selected PTX-down proliferation-associated genes and PTX-up adaptive or stress-associated genes are labeled. **(B)** Heatmap of representative PTX-down and PTX-up genes across DX and PTX malignant-neuroblast pseudo-bulk samples. Gene expression values are shown as row-scaled z-scores. **(C)** Hallmark enrichment analysis of PTX-down and PTX-up gene sets. PTX-down genes were enriched for E2F targets, G2/M checkpoint, MYC targets, DNA repair, mitotic spindle, mTORC1 signaling, and glycolysis, whereas PTX-up genes were enriched for TNFA signaling via NFKB, hypoxia, inflammatory response, IL6/JAK/STAT3 signaling, apoptosis, heme metabolism, TGF-beta signaling, epithelial-mesenchymal transition, KRAS signaling, and complement pathways.

Heatmap visualization of representative PTX-regulated genes confirmed a reciprocal transcriptional pattern between DX and PTX pseudo-bulk profiles. PTX-down genes were preferentially expressed in DX malignant-neuroblast samples and reduced in PTX samples, whereas PTX-up genes showed the opposite pattern, with relatively higher expression in PTX profiles (Figure 2B). These results indicate that PTX malignant-neuroblast profiles are characterized by lower expression of proliferative tumor-cell programs and relative enrichment of transcriptional features associated with residual adaptive states.

To functionally characterize these therapy-regulated gene sets, we performed Hallmark pathway enrichment analysis. PTX-down genes were strongly enriched for E2F targets, G2/M checkpoint, MYC targets, DNA repair, mitotic spindle, mTORC1 signaling, and glycolysis, consistent with suppression of a proliferative-replication axis after treatment. By contrast, PTX-up genes were enriched for TNFA signaling via NFKB, hypoxia, inflammatory response, IL6/JAK/STAT3 signaling, apoptosis, heme metabolism, TGF-beta signaling, epithelial-mesenchymal transition, KRAS signaling, and complement pathways (Figure 2C). Together, these findings indicate that PTX malignant-neuroblast profiles differ from matched DX profiles through reduced E2F/G2M/MYC-associated proliferative programs and relative enrichment of stress-adaptive and inflammatory transcriptional states.

### PTX-down proliferation score predicts adverse OS and EFS in GSE62564

Given that the PTX-down gene set represented a chemotherapy-suppressed proliferative-replication program, we next asked whether baseline activity of this program in diagnostic bulk tumors was associated with clinical outcome. For each bulk RNA-seq cohort, expression values of PTX-down genes were standardized gene-wise within the cohort, and a sample-level PTX-down proliferation score was calculated as the mean z-score across PTX-down genes. Accordingly, the PTX-down score quantifies diagnostic activity of a malignant-neuroblast program that becomes suppressed after induction therapy. Patients were then stratified into PTX-down high and low groups using the cohort-specific median score (Figure 3A).

**FIGURE 3:**
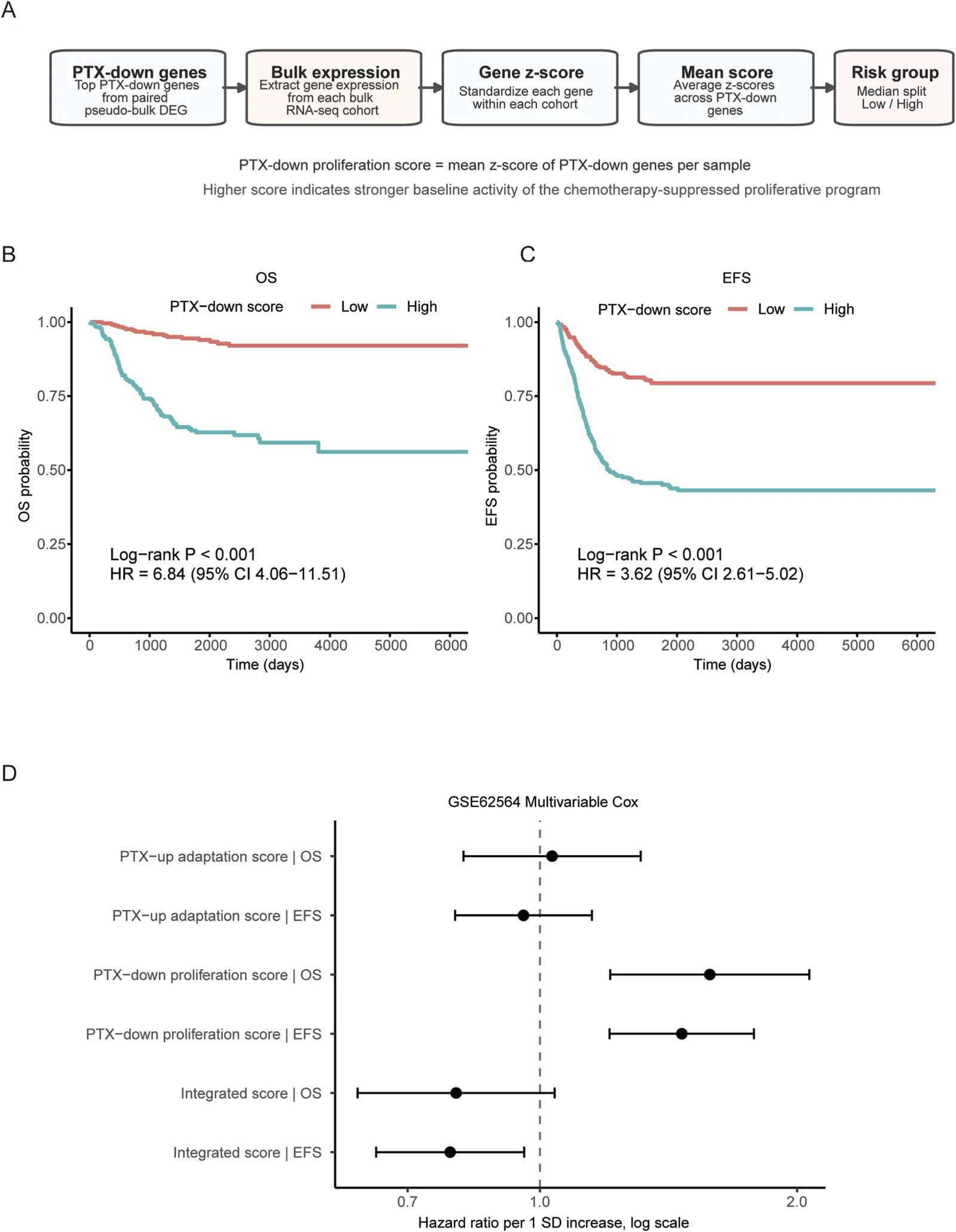
PTX-down proliferation score predicts adverse OS and EFS in GSE62564. **(A)** Schematic illustrating construction of the PTX-down proliferation score. PTX-down genes identified from paired malignant-neuroblast pseudo-bulk analysis were extracted from each bulk RNA-seq cohort, standardized gene-wise within each cohort, and averaged to generate a sample-level PTX-down proliferation score. Patients were dichotomized by the cohort-specific median score. **(B,C)** Kaplan-Meier curves showing overall survival (OS) and event-free survival (EFS) stratified by median PTX-down proliferation score in GSE62564. **(D)** Multivariable Cox analysis of PTX-derived scores for OS and EFS in GSE62564. Hazard ratios are shown per 1 standard deviation increase in each score. Models were adjusted for age, COG-defined high-risk classification, MYCN status, and INSS stage.

In the primary validation cohort GSE62564, patients with high PTX-down proliferation score showed markedly worse overall survival (OS) than those with low score. The high-score group had a hazard ratio (HR) of 6.84 for OS, with a 95% confidence interval (CI) of 4.06–11.51, and the log-rank test was highly significant (P < 0.001) (Figure 3B). A similar pattern was observed for event-free survival (EFS), where the PTX-down high group showed significantly inferior outcome compared with the low-score group (HR = 3.62, 95% CI 2.61–5.02; log-rank P < 0.001) (Figure 3C). These results indicate that stronger baseline activity of the chemotherapy-suppressed proliferative program is associated with adverse survival in neuroblastoma.

To further determine whether this association was independent of established clinical risk factors, we performed multivariable Cox regression analysis in GSE62564. After adjustment for age, COG-defined high-risk classification, MYCN status, and INSS stage, the PTX-down proliferation score remained associated with increased risk for both OS and EFS. By contrast, the PTX-up adaptation score showed weaker or non-significant associations after clinical adjustment, and the integrated score showed an opposite direction for selected endpoints (Figure 3D). These findings support the PTX-down proliferation score as the dominant adverse prognostic component among the therapy-derived scores.

Sensitivity analyses supported the robustness of the PTX-down proliferation score to alternative gene-set cutoffs and ranking strategies. When the PTX-down score was recalculated using the top 200, top 300, or top 400 PTX-down genes ranked by the composite score, the score remained strongly associated with adverse OS and EFS in GSE62564. The corresponding HRs per 1-SD increase were 3.16, 3.11, and 3.08 for OS, and 2.16, 2.14, and 2.13 for EFS, respectively. Similar results were obtained when PTX-regulated genes were ranked by absolute log2 fold change alone rather than by the composite score. Across external cohorts, the adverse direction of the PTX-down score was generally preserved, although the magnitude and statistical significance varied by dataset. Alternative PTX-down scores were highly correlated with the primary top300 composite-score-based PTX-down score across cohorts, with Spearman correlation coefficients ranging from 0.969 to 1.000. Hallmark enrichment analyses also consistently identified E2F targets, G2/M checkpoint, mitotic spindle, DNA repair, and MYC-related proliferative-replication pathways across alternative specifications. These findings indicate that the biological and prognostic interpretation of the PTX-down score was not dependent on the specific top 300 cutoff or the primary composite ranking strategy (Supplementary Table S8).

In addition, random gene-set null analysis in GSE62564 showed that the primary PTX-down top300 score was located far beyond the random null distribution generated from 1,000 randomly sampled gene sets of the same size for both OS and EFS (Supplementary Figure S1; Supplementary Table S8E,F). The empirical one-sided P value was <0.001 for both endpoints, supporting the specificity of the PTX-down program relative to arbitrary gene-set selection.

### PTX-down proliferation score improves prognostic discrimination beyond clinical risk variables

We next evaluated whether the PTX-down proliferation score could improve prognostic discrimination beyond established clinical models. Two baseline models were considered in GSE62564: a COG model based on COG-defined high-risk classification, and a conventional clinical model incorporating age, MYCN status, and INSS stage. We compared each baseline model with its corresponding expanded model after adding the PTX-down proliferation score.

For both OS and EFS, adding the PTX-down proliferation score increased the C-index of the COG model and the conventional clinical model (Figure 4A). This suggests that the PTX-down score provides additional prognostic information beyond standard clinical stratification. The improvement was particularly evident when the score was added to the COG high-risk model, consistent with the ability of the PTX-down score to capture transcriptional heterogeneity within clinically defined risk groups.

**FIGURE 4:**
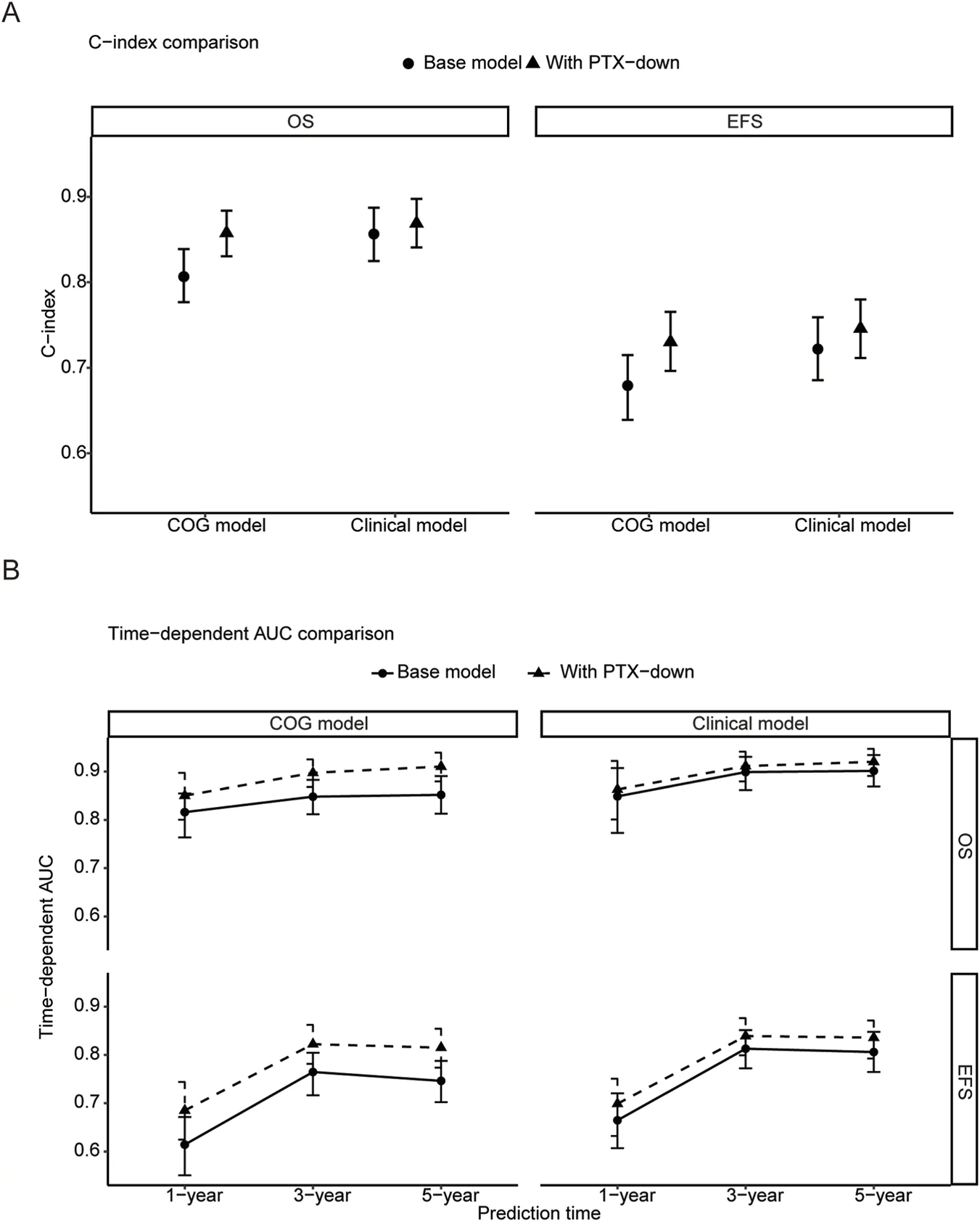
PTX-down proliferation score improves prognostic discrimination beyond clinical risk variables. **(A)** C-index comparison between baseline clinical models and corresponding expanded models after adding the PTX-down proliferation score in GSE62564. Baseline models included the COG-defined high-risk model and the conventional clinical model based on age, MYCN status, and INSS stage. Points indicate C-index estimates, and error bars indicate bootstrap 95% confidence intervals. **(B)** Time-dependent AUC comparison at 1, 3, and 5 years for OS and EFS in GSE62564. Base models and corresponding models plus the PTX-down proliferation score are shown. Points indicate time-dependent AUC estimates, and error bars indicate bootstrap 95% confidence intervals. Numerical values, confidence intervals, and model-performance differences are provided in Supplementary Table S9.

Time-dependent AUC analysis further supported the incremental value of the PTX-down score. Across 1-, 3-, and 5-year prediction intervals, adding the PTX-down proliferation score consistently improved AUC for both OS and EFS. In the COG model, AUC improvements ranged from +0.035 to +0.058 for OS and from +0.059 to +0.070 for EFS. In the conventional clinical model, AUC improvements were smaller but stable, ranging from +0.020 to +0.025 for OS and from +0.026 to +0.029 for EFS (Figure 4B). Together, these analyses demonstrate that the PTX-down proliferation score not only stratifies survival but also improves prognostic discrimination when incorporated into clinical risk models.

### Cross-cohort validation links PTX-down proliferation score to adverse OS and high-risk clinical biology

To assess generalizability, we evaluated the association between PTX-down proliferation score and OS across four bulk RNA-seq cohorts, including the primary cohort GSE62564 and three additional cohorts (GSE181559, TARGET-NBL, and fwr144). In each cohort, the PTX-down proliferation score was calculated using the same gene-wise z-score strategy, and Cox regression was performed using the score as a continuous variable.

Across all four cohorts, higher PTX-down score showed a consistent adverse direction for OS, with HR values greater than 1. Random-effects meta-analysis confirmed a significant pooled association between higher PTX-down score and worse OS, with a pooled HR of 1.83, 95% CI 1.20–2.80, and P = 0.005 (Figure 5A). Between-cohort heterogeneity was substantial (I^2^ = 87.7%), likely reflecting differences in cohort composition, treatment background, clinical annotation, and preprocessing. Nevertheless, the consistent directionality across datasets supports the PTX-down score as an adverse prognostic transcriptional program.

**FIGURE 5:**
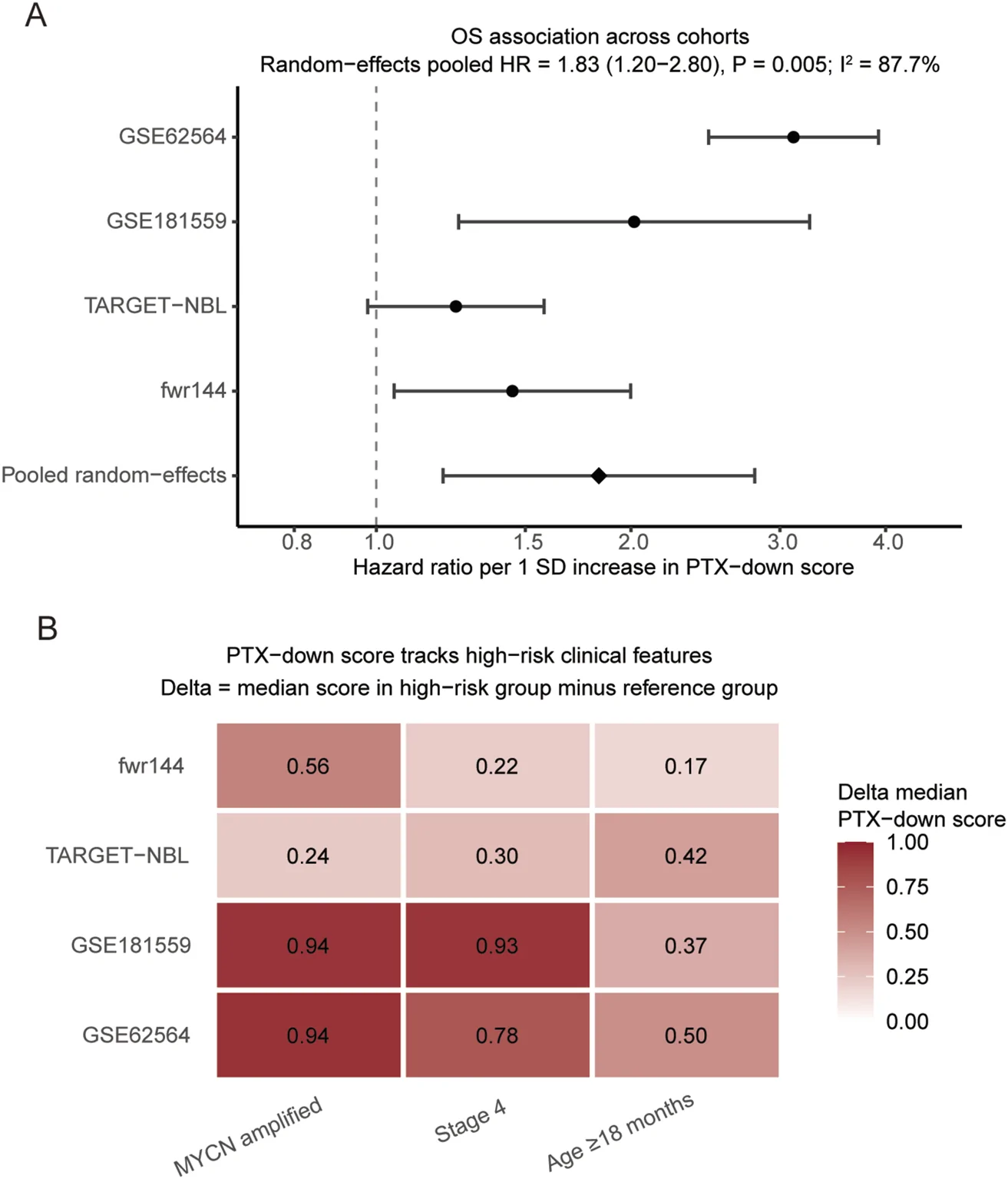
Cross-cohort validation links the PTX-down proliferation score to adverse OS and high-risk clinical biology. **(A)** Forest plot showing the association between PTX-down proliferation score and OS across four bulk RNA-seq cohorts. Hazard ratios are shown per 1 standard deviation increase in PTX-down score. The pooled estimate was calculated using a random-effects model. **(B)** Heatmap showing differences in median PTX-down proliferation score between high-risk and reference clinical subgroups across bulk RNA-seq cohorts. Positive values indicate higher PTX-down score in tumors with MYCN amplification, stage 4 disease, or age 18 months or older.

We next examined whether the PTX-down proliferation score was associated with established high-risk clinical and biological features across cohorts. In all four bulk RNA-seq cohorts, the score was higher in tumors with MYCN amplification, stage 4 disease, and age ≥ 18 months compared with their respective reference groups (Figure 5B). The magnitude of these differences varied across datasets, but the direction was consistently positive. These findings suggest that the PTX-down proliferation score captures transcriptional features aligned with known high-risk neuroblastoma biology, providing a potential explanation for its association with adverse OS.

### PTX-down score reflects an E2F/G2M/MYC proliferative axis in diagnostic tumors

To further interpret the biological meaning of the PTX-down proliferation score in diagnostic bulk tumors, we examined representative core genes from the PTX-down program in GSE62564. Tumors were ordered by PTX-down score, and expression patterns of genes related to the E2F/MYC axis, DNA replication, and cell-cycle/mitotic regulation were visualized. Samples with higher PTX-down score showed coordinated upregulation of core proliferation-associated genes, including E2F1, E2F2, MYBL2, UHRF1, PCNA, MCM2, MCM4, MCM5, MCM6, MCM7, MCM10, CDC45, EXO1, MKI67, TOP2A, AURKB, BIRC5, CDK1, CCNB1, CCNB2, CENPF, KIF11, and KIF18B (Figure 6A). These high-score tumors were also enriched for clinical annotations corresponding to COG-defined high-risk status, MYCN amplification, and INSS stage 4, linking the molecular score to aggressive neuroblastoma phenotypes.

**FIGURE 6:**
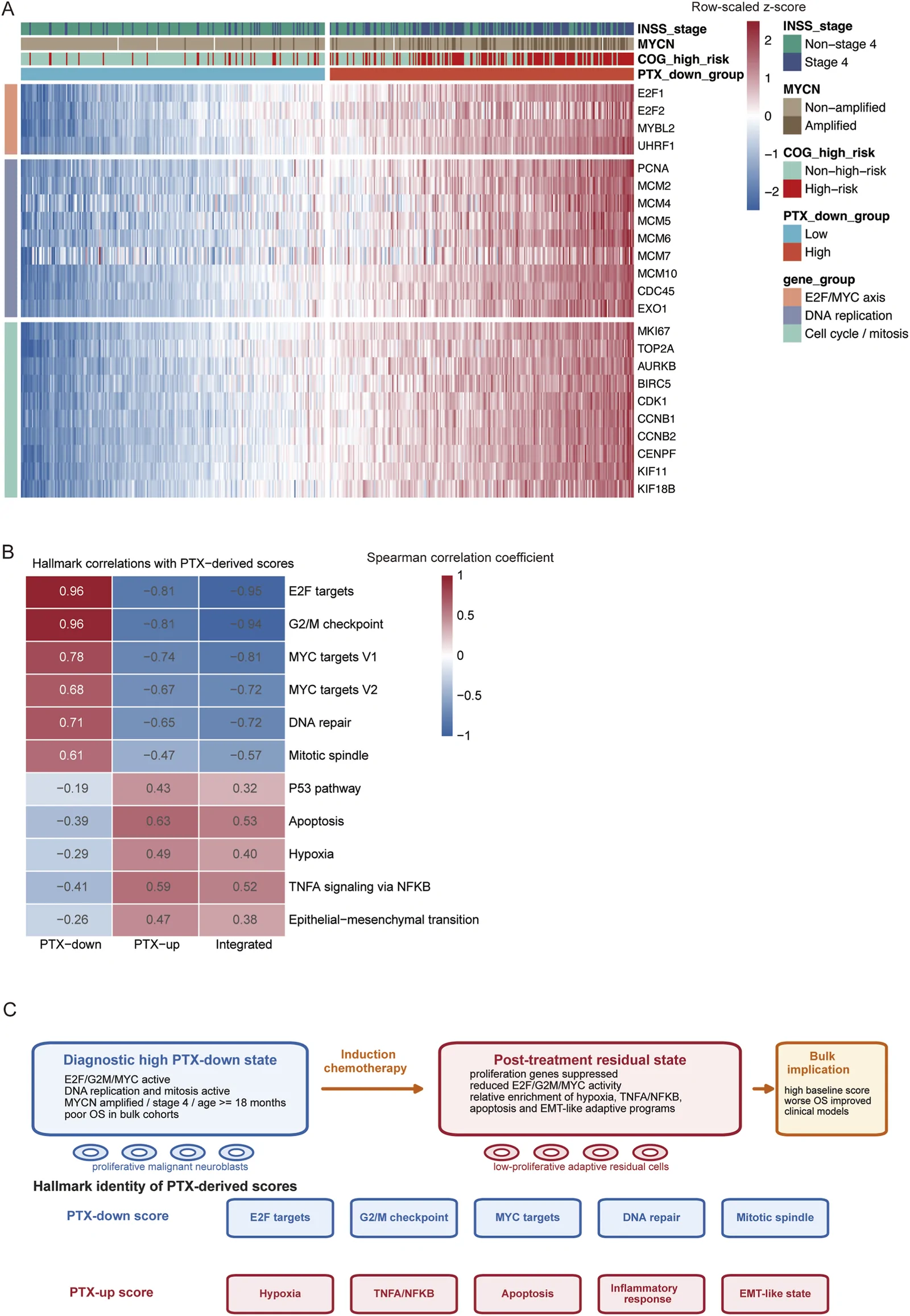
Biological bridge defining the PTX-down score as an E2F/G2M/MYC proliferative axis. **(A)** Heatmap of representative PTX-down core genes in GSE62564 tumors ordered by PTX-down proliferation score. Gene expression values are shown as row-scaled z-scores, with annotations for PTX-down score group, COG-defined high-risk status, MYCN status, INSS stage, and gene group. **(B)** Spearman correlations between PTX-derived scores and selected Hallmark pathway activities in GSE62564. The PTX-down score was strongly correlated with E2F targets, G2/M checkpoint, MYC targets, DNA repair, and mitotic spindle programs, whereas PTX-up and integrated scores showed reciprocal associations. **(C)** Therapy-informed framework linking a chemotherapy-suppressed malignant-neuroblast proliferative-replication program to baseline high-risk biology, clinical model improvement, and adverse outcome in diagnostic bulk tumors.

Pathway-level correlation analysis further confirmed that the PTX-down score represents a proliferative-replication axis. In GSE62564, the PTX-down score showed strong positive correlations with Hallmark activities of E2F targets, G2/M checkpoint, MYC targets, DNA repair, and mitotic spindle. In contrast, the PTX-up adaptation score showed reciprocal negative correlations with these proliferative pathways and positive correlations with pathways such as apoptosis, hypoxia, TNFA signaling via NFKB, and epithelial-mesenchymal transition (Figure 6B). The integrated score showed a pattern closer to the adaptive state, further supporting the opposing biological directions of the PTX-down and PTX-up programs.

Together, these findings support a model in which induction chemotherapy suppresses an E2F/G2M/MYC-driven proliferative-replication program in residual malignant neuroblasts. However, when this chemotherapy-suppressed program is highly active at diagnosis, it marks tumors with more aggressive baseline biology, including MYCN amplification, stage 4 disease, older age at diagnosis, and poor OS across bulk cohorts (Figure 6C). Thus, the PTX-down proliferation score functions not simply as a statistical survival signature, but as a biologically interpretable measure of a treatment-suppressed proliferative axis linked to high-risk neuroblastoma biology and adverse outcome.

## Discussion

This study links therapy-associated single-cell tumor states with diagnostic risk stratification in neuroblastoma. By integrating longitudinal single-cell/single-nucleus RNA-seq data with multiple diagnostic bulk RNA-seq cohorts, we identified a PTX-down malignant-neuroblast program characterized by E2F targets, G2/M checkpoint, MYC targets, DNA repair, mitotic spindle, and other proliferation-associated pathways.

When projected onto diagnostic bulk tumors, this PTX-down program yielded a proliferation score associated with adverse OS and EFS in GSE62564, improved prognostic discrimination beyond clinical risk variables, and showed adverse OS association across external cohorts. These findings suggest that high baseline activity of a therapy-reduced proliferative-replication program captures aggressive neuroblastoma biology.

A major implication of this work is that therapy-associated single-cell programs can be repurposed to interpret baseline tumor risk. Unlike diagnostic bulk-derived signatures selected solely for survival association, the PTX-down score was first defined as a longitudinal therapy-regulated malignant-neuroblast program and only then evaluated for prognostic relevance in independent diagnostic cohorts. This design gives the score a therapy-informed biological context: it reflects a proliferative-replication program that is reduced after induction chemotherapy but whose high baseline activity marks tumors with adverse clinical behavior. In this respect, the score should be interpreted less as a conventional prognostic signature and more as a quantitative readout of a treatment-sensitive malignant-neuroblast state.

Several bulk RNA-seq prognostic signatures have previously been proposed for neuroblastoma, including ferroptosis-related, immune- or microenvironment-associated, and cancer-associated fibroblast-related models developed using cohorts such as GSE62564 and TARGET-NBL (Chen et al., 2022; Aierken et al., 2025; Zhao et al., 2025). These studies have been valuable for risk stratification and for highlighting biological processes such as redox regulation, immune activity, stromal remodeling, and tumor-microenvironment interactions. However, most of these signatures are derived directly from diagnostic bulk tumor profiles by selecting genes associated with survival or predefined biological categories. The PTX-down proliferation score differs conceptually from these approaches. It was first defined from paired longitudinal malignant-neuroblast pseudo-bulk analysis as a program reduced after induction therapy, and was subsequently projected onto diagnostic bulk transcriptomes. Thus, the main novelty of the PTX-down score is not that it represents another bulk-derived prognostic model, but that it provides a therapy-informed readout of a tumor-cell-intrinsic proliferative-replication state. This state is aligned with the proliferative and metabolically active neoplastic programs described in recent longitudinal single-cell multi-omic studies, but our analysis further connects this treatment-regulated malignant-neuroblast program to diagnostic bulk risk, clinical model improvement, and cross-cohort adverse OS association. Accordingly, the PTX-down score should be interpreted as complementary to prior ferroptosis-, immune-, or cancer-associated fibroblast (CAF)-related signatures, rather than as a replacement for them.

The PTX-down program identified here is dominated by canonical cell-cycle and DNA replication features, including E2F/G2M/MYC-associated signaling, MCM family genes, mitotic regulators, and DNA repair-related components. These programs are central to malignant proliferation and are consistent with the aggressive biology of high-risk neuroblastoma, particularly in MYCN-amplified and advanced-stage disease. The observation that the PTX-down score was higher in tumors with MYCN amplification, stage 4 disease, and age 18 months or older further supports its alignment with established high-risk clinical biology. Beyond its survival association, the PTX-down score improved model discrimination over both COG-based and conventional clinical models, indicating that it captures transcriptional heterogeneity not resolved by standard risk variables.

These findings are consistent with emerging single-cell and spatial multi-omic studies showing that neuroblastoma is characterized by developmental plasticity, intratumoral heterogeneity, and epigenetically primed state transitions (Jansky et al., 2021; Xu et al., 2025; Yu et al., 2025). Within this framework, the PTX-down score may be viewed as a therapy-informed readout of a proliferative malignant-neuroblast state that is clinically adverse when dominant at diagnosis, rather than merely as a bulk-derived prognostic score. Thus, the present analysis extends recent longitudinal atlas work by converting a treatment-regulated malignant-cell state into a diagnostic bulk readout with prognostic and biological interpretability.

Another notable finding is the reciprocal relationship between PTX-down and PTX-up programs. While PTX-down genes reflected proliferative and replication-associated biology, PTX-up genes were enriched for hypoxia, inflammatory signaling, apoptosis, TGF-beta/EMT-related features, and other adaptive-state pathways. This pattern suggests that induction therapy reshapes malignant neuroblasts by suppressing highly proliferative programs while relatively enriching residual states with stress-adaptive features. Such reciprocal remodeling is compatible with the concept of drug-tolerant persister states in which residual malignant cells suppress MYC/MYCN-linked proliferation programs and activate inflammatory or stress-response pathways, rather than maintaining the original high-replication state. In this framework, the PTX-down score is not a marker of post-treatment persistence *per se*; rather, it reflects the degree to which a diagnostic tumor is driven by the proliferative program that is subsequently suppressed by therapy.

The enrichment of DNA replication, mitotic, and E2F/MYC-related programs in PTX-down high tumors also raises a testable therapeutic hypothesis. Recent preclinical studies have highlighted MYCN/E2F-linked transcriptional dependencies and replication-stress-associated vulnerabilities in neuroblastoma, including strategies that perturb MYCN transcriptional control or ribonucleotide reductase/CHK1-mediated replication-stress buffering. Although the present study does not establish therapeutic sensitivity, the convergence of E2F/MYC activity, DNA replication genes, and adverse outcome suggests that PTX-down high tumors may warrant future functional evaluation for vulnerabilities along the proliferative-replication and replication-stress axes (Pandher et al., 2025; Nelen et al., 2026).

The cross-cohort analyses support the generalizability of the PTX-down score while also revealing important heterogeneity. Across four bulk RNA-seq cohorts, higher PTX-down score was consistently associated with adverse OS, and the random-effects meta-analysis confirmed a significant pooled effect. However, heterogeneity was substantial, likely reflecting differences in cohort composition, expression processing, clinical annotation, treatment background, follow-up duration, and sample size. EFS associations were directionally consistent but weaker than OS, suggesting that endpoint definitions and cohort-specific event structures may influence the observed effect size. Therefore, the PTX-down score should be interpreted as a biologically informed prognostic marker rather than a finalized clinical assay.

Future functional studies will be required to determine whether PTX-down core genes directly contribute to proliferative recovery, chemotherapy response, or relapse-associated behavior in neuroblastoma. Candidate genes from the E2F/G2M/MYC and DNA replication modules, including E2F1, MYBL2, PCNA, MCM family members, CDC45, EXO1, TOP2A, AURKB, BIRC5, and CDK1, could be prioritized for perturbation experiments in neuroblastoma cell lines, patient-derived organoids, or xenograft-derived models. Loss- and gain-of-function experiments could be combined with conventional chemotherapy exposure to assess effects on proliferation, cell-cycle progression, DNA damage accumulation, apoptosis, and post-treatment regrowth. In parallel, PTX-down-high and PTX-down-low models could be compared for sensitivity to agents targeting replication-stress response or cell-cycle checkpoint pathways. Such experiments would be necessary to determine whether the transcriptomic associations observed in this study reflect actionable biological dependencies.

This study has several strengths. First, the discovery analysis was based on longitudinal single-cell data, enabling direct comparison of malignant neuroblasts before and after treatment. Second, pseudo-bulk aggregation allowed donor-aware differential expression analysis while reducing single-cell sparsity. Third, the PTX-down program was validated in multiple diagnostic bulk RNA-seq cohorts and evaluated at several levels, including survival stratification, multivariable Cox regression, model discrimination, meta-analysis, clinical feature association, and pathway-level biological interpretation. Together, these analyses provide convergent evidence that the PTX-down score captures a clinically relevant proliferative axis.

Several limitations should also be acknowledged. First, the single-cell discovery dataset, although longitudinal and well suited to therapy-associated state analysis, remains limited by sample availability, treatment timing, and the structure of paired DX/PTX specimens. Treatment regimens, sampling intervals, tumor purity, and residual disease composition may influence the observed PTX-associated programs. Second, malignant neuroblast identification and pseudo-bulk aggregation depend on the quality of cell-type annotation and donor-level metadata. Third, the bulk validation cohorts differ in expression units, clinical variables, endpoint definitions, treatment background, and follow-up duration, which may contribute to heterogeneity across datasets. In addition, GSE62564 served both as the primary validation cohort and as one of the four cohorts in the cross-cohort meta-analysis, which may upwardly bias the pooled estimate; the meta-analysis should therefore be interpreted as supportive rather than fully independent external validation. Notably, the multivariable Cox model adjusted simultaneously for COG-defined high-risk classification, MYCN status, INSS stage, and age, which exhibit partial collinearity because COG risk is itself derived from these variables; the adjusted hazard ratios should therefore be interpreted as evidence of independence beyond conventional risk stratification rather than as fully orthogonal effect estimates. Fourth, the PTX-down score was evaluated retrospectively using public datasets and should not yet be regarded as a finalized clinical assay. Prospective validation in uniformly treated high-risk neuroblastoma cohorts will be required before clinical translation. Finally, this study is primarily transcriptomic and computational. Functional experiments are needed to determine whether specific PTX-down core genes directly mediate chemotherapy sensitivity, proliferative recovery, relapse risk, or vulnerability to replication-stress-targeted strategies.

Despite these limitations, our findings provide a conceptual framework for translating therapy-associated single-cell remodeling into diagnostic bulk prognostic interpretation. The PTX-down proliferation score connects a chemotherapy-suppressed malignant-neuroblast program with high-risk baseline tumor biology, adverse OS, and improved prognostic model performance. Rather than representing a purely statistical signature, the score reflects an interpretable E2F/G2M/MYC-driven proliferative-replication axis. This therapy-informed perspective suggests that longitudinally observed state remodeling can be leveraged to interpret baseline high-risk biology and to guide future studies of residual disease biology, proliferative vulnerability, and treatment-adaptive states in high-risk neuroblastoma.

## Conclusion

Longitudinal single-cell analysis identified a chemotherapy-suppressed proliferative-replication program in malignant neuroblasts. When projected onto diagnostic bulk RNA-seq cohorts, baseline activity of this PTX-down program predicted adverse survival, improved prognostic discrimination beyond clinical risk variables, and tracked established high-risk biological features. These findings define the PTX-down proliferation score as a biologically interpretable E2F/G2M/MYC-driven axis linking chemotherapy-suppressed tumor-cell remodeling to high-risk neuroblastoma biology and adverse prognosis.

## Data Availability

The datasets analyzed in this study were obtained from publicly available resources, including the longitudinal single-cell/single-nucleus RNA-seq neuroblastoma atlas and bulk RNA-seq cohorts GSE62564, GSE181559, TARGET-NBL, and fwr144. The longitudinal single-cell atlas is available through public repositories associated with the original study, including CELLxGENE/HTAN-linked resources; the CELLxGENE dataset identifier used in this study was cee845e3-ec04-4781-9e2a-28734bb4f7ba. Processed results supporting the main figures are provided in the Supplementary Tables. Analysis code used in this study will be made publicly available on GitHub upon publication. Additional intermediate files are available from the corresponding author upon reasonable request.
